# Impact of vertebrate communities on *Ixodes ricinus*-borne disease risk in forest areas

**DOI:** 10.1186/s13071-019-3700-8

**Published:** 2019-09-06

**Authors:** Katsuhisa Takumi, Hein Sprong, Tim R. Hofmeester

**Affiliations:** 1 Centre for Zoonoses and Environmental Microbiology, Centre for Infectious Disease Control, National Institute for Public Health and the Environment (RIVM), Bilthoven, The Netherlands; 2 Resource Ecology Group, Wageningen University, Wageningen, The Netherlands; 3 Present Address: Department of Wildlife, Fish, and Environmental Studies, Swedish University of Agricultural Sciences, Skogsmarksgränd 7, 907 36 Umeå, Sweden

**Keywords:** *Ixodes ricinus*, *Anaplasma phagocytophilum*, *Borrelia burgdorferi* (*s.l.*), *Neoehrlichia mikurensis*, *Borrelia miyamotoi*, Vector-borne disease, Lyme borreliosis, Transmission dynamics

## Abstract

**Background:**

The density of questing ticks infected with tick-borne pathogens is an important parameter that determines tick-borne disease risk. An important factor determining this density is the availability of different wildlife species as hosts for ticks and their pathogens. Here, we investigated how wildlife communities contribute to tick-borne disease risk. The density of *Ixodes ricinus* nymphs infected with *Borrelia burgdorferi* (*sensu lato*), *Borrelia miyamotoi*, *Neoehrlichia mikurensis* and *Anaplasma phagocytophilum* among 19 forest sites were correlated to the encounter probability of different vertebrate hosts, determined by encounter rates as measured by (camera) trapping and mathematical modeling.

**Result:**

We found that the density of any tick life stage was proportional to the encounter probability of ungulates. Moreover, the density of nymphs decreased with the encounter probability of hare, rabbit and red fox. The density of nymphs infected with the transovarially-transmitted *B. miyamotoi* increased with the density of questing nymphs and the encounter probability of bank vole. The density of nymphs infected with all other pathogens increased with the encounter probability of competent hosts: bank vole for *Borrelia afzelii* and *N. mikurensis*, ungulates for *A. phagocytophilum* and blackbird for *Borrelia garinii* and *Borrelia valaisiana*. The negative relationship we found was a decrease in the density of nymphs infected with *B. garinii* and *B. valaisiana* with the encounter probability of wood mouse.

**Conclusions:**

Only a few animal species drive the densities of infected nymphs in forested areas. There, foxes and leporids have negative effects on tick abundance, and consequently on the density of infected nymphs. The abundance of competent hosts generally drives the abundances of their tick-borne pathogen. A dilution effect was only observed for bird-associated Lyme spirochetes.
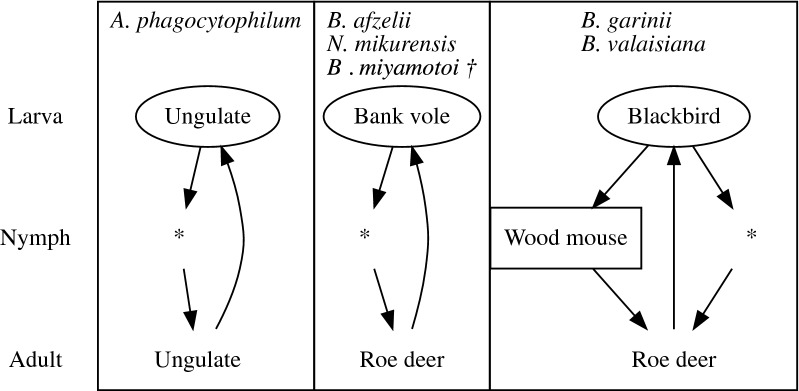

## Background

Lyme borreliosis poses serious health concerns in Europe as well as in North America [1, 2]. Furthermore, diseases caused by other tick-borne pathogens (TBP) such as *Borrelia miyamotoi*, *Neoehrlichia mikurensis* and *Anaplasma phagocytophilum* are emerging or being (re)discovered [2, 3]. Understanding which factors drive population densities of ticks and the transmission cycles of their pathogens are important steps in assessing disease risk and formulating possible intervention strategies [4–7].

Infection by any TBP in humans is preceded by a bite of an infected tick. About two-thirds of tick bites reported are nymphal *Ixodes ricinus* ticks [8–11]. Therefore, the density of questing nymphs infected with TBPs is often referred to as an important ecological parameter that, together with the level of human exposure, determines tick-borne disease risk [12, 13].

Density of questing, infected nymphal ticks (DIN) can be calculated by multiplying the nymphal infection prevalence (NIP) by the density of questing nymphal *I. ricinus* (DON). Both DON [14, 15] and NIP vary widely, both spatially and temporally, for different TBPs, *B. burgdorferi* (*s.l.*) [15], *B. miyamotoi* [16], *N. mikurensis* [14] and *A. phagocytophilum* [17], resulting in a large variation in a common measure of public health risk, DIN. These variations have partly been attributed to environmental factors such as differences in weather conditions [1, 18] and habitat characteristics [19–21].

However, there is growing evidence that differences in host availability also play a big role [22]. This is because wildlife and free-ranging domestic animals act as feeding and propagation hosts for *I. ricinus* and as reservoir hosts for TBPs [23]. For instance, the presence of deer correlates to a high value in local DON, while DON is low in the absence of deer [24], which is likely due to the importance of deer as feeding host for adult *I. ricinus* [23]. It is theorized that the infection prevalence of TBPs in ticks in the USA increases with the abundance of a vertebrate species that is susceptible for colonization by the pathogen (competent host) [25]. Small mammals, particularly rodents, are considered to be competent hosts for *B. afzelii* [26, 27], *N. mikurensis* and *B. miyamotoi* [28], while songbirds are competent hosts for *B. garinii* and *B. valaisiana* [29, 30] and ungulates are considered to be competent hosts for *A. phagocytophilum* ecotypes I and II [17, 31]. The number of studies correlating the densities of these hosts with infection prevalence of their respective pathogens is, however, limited to a situation in the USA [22] and one other situation in Europe [32].

At the same time, vertebrate communities might decrease the density of infected *I. ricinus* with several, often poorly understood, mechanisms [32–34]. An example is the dilution effect hypothesis, where diluting the abundance of competent hosts with non-competent hosts will reduce the probability of ticks feeding on transmission-competent hosts and consequently decrease the infection prevalence of pathogens in ticks [35]. Although the original study stating the dilution effect hypothesis considered Lyme disease in North America [35], there is insufficient evidence about the validity of the dilution effect hypothesis for Lyme borreliosis, particularly in European settings, where the hypothesis might only be valid only under specific conditions or for specific TBPs [36–38].

Here, we investigated how local communities of vertebrates are contributing to the densities of questing infected ticks in Dutch forests testing for both amplification and dilution effects of specific host species or taxonomic groups. We used a combination of camera trapping and live trapping to quantify the availability of hosts to ticks at 19 forested sites in the Netherlands. As the sequence of events in which individual animals of a specific host species arrive to a field of view of the camera lens or are caught in a live trap is expected to follow a Poisson process [39], we could use these encounter rates to estimate the encounter probability for each host species. First, we linked the density of the three tick-instar stages to the encounter probability of four ungulate species: roe deer (*Capreolus capreolus*), fallow deer (*Dama dama*), red deer (*Cervus elaphus*) and wild boar (*Sus scrofa*) as important amplification hosts [23]. Secondly, we tested for an association between questing tick densities and the encounter probability of red fox (*Vulpes vulpes*) and leporids, i.e. European hare (*Lepus europaeus*) and rabbit (*Oryctolagus cuniculus*). Thirdly, we linked the density of questing infected nymphs for five species of TBPs to nymphal density and the encounter probability of different host species. Fourthly, we analyzed the presence of two TBPs in a single nymphal tick (co-infection) to test for independence of individual TBP life-cycles. It is possible to determine a component of the vertebrate community as the driver of DIN for a particular pathogen by modelling molecular detection of the five species of TBPs in questing nymphal ticks in the same forest.

## Methods

### Tick collection and identification

Data on hosts, ticks and tick-borne pathogens (TBPs) were collected at 19 sites located in forested areas in the Netherlands in 2013 and 2014 (Additional file 1: Figure S1). Details on study locations (including an exclosure that was not taken into account in this study) and data collection were described previously [32]. Briefly, ticks were collected six times at each site, once every four weeks between April and September, by cotton-flagging of twenty 10-m transects using a 1 m^2^ cotton cloth, totaling 1200 m^2^. Flagging ticks was only performed with optimal conditions: on dry days, with air temperature > 10 °C and in dry vegetation < 60 cm high. During each session, flagging was performed within five days in all sites to minimize variation in weather conditions. All ticks were collected in Eppendorf tubes and stored at − 20 °C until pathogen analysis. Upon arrival in the laboratory, the ticks were identified by an experienced technician using morphological keys as described in Arthur [40] and Hillyard [41]. Only *I. ricinus* nymphs were used for further analysis.

### Pathogen identification and prevalence

DNA extraction from the individual questing ticks was achieved by alkaline lysis in ammonium hydroxide [42]. For the detection of *B. burgdorferi* (*s.l.*) DNA, a duplex qPCR was used, based on the detection of fragments of the outer surface protein A (ospA) and flagellin genes [43]. A conventional PCR assay, targeting the 5S-23S intergenic spacer region (IGS), was performed for *B. burgdorferi* (*s.l.*) genospecies identification [44]. Conventional PCR assays were carried out in a Px2 thermal cycler (Thermo Electron Corporation, Breda, Netherlands) and visualized on a 2% agarose gel. Both strands of PCR products were sequenced by BaseClear (Leiden, Netherlands), according to the company’s protocol and using the same forward and reverse primers as in the conventional PCR. BLAST analyses and an in-house molecular epidemiological database (Bionumerics 7.1 Applied Math, Sint-Martens-Latem, Belgium) were used to identify *B. burgdorferi* (*s.l.*) genospecies after trimming and manual cleaning of newly obtained DNA sequences. The in-house molecular epidemiological database contains more than 10,000 IGS-sequences from (field) isolates and GenBank [44, 45]. For detection of *B. miyamotoi*, a qPCR assay was used that targets a region of the flagellin gene, specific for *B. miyamotoi* [46]. For detection of *A. phagocytophilum* and *N. mikurensis* DNA, a duplex qPCR assay was used, as described by Jahfari et al. [17, 47]. This qPCR assay targets specific regions of the major surface protein 2 gene (*msp2*) for *A. phagocytophilum* and a heat-shock protein gene (groEL) for *N. mikurensis*. All qPCR runs were carried out in a final volume of 20 μl, containing IQ Multiplex Powermix (Bio-Rad Laboratories, Hercules, CA, USA), 400 nM primers and hydrolysis probes and 3 μl of DNA template. Conditions for PCR amplification were as follows: 95 °C for 5 min, 60 cycles at 95 °C for 5 s and 60 °C for 35 s, followed by a final incubation step at 37 °C for 20 s. We carried out qPCR assays on a LightCycler 480 instrument (Roche Diagnostics Nederland B.V, Almere, Netherlands) and analysis was performed by the instrument’s software (release 1.5.1.62). Quantification cycle (Cq) values were calculated using the second derivative method. For each qPCR run, three positive controls, two negative controls, and two blank samples were included. We determined nymphal infection prevalence (NIP) as the fraction of a pathogen’s presence in a collection of nymphal ticks randomly selected for the molecular test.

### Host identification and quantification

We quantified the availability of vertebrate hosts to ticks by estimating the encounter rate of different species based on camera-trap and live-trap data [32]. We use the term encounter rate synonymous with passage rate. Using the reasoning in Lucas et al. [48] and Hofmeester et al. [49], the contact rate between a vertebrate host and a camera trap ($R_{\text{cam}}$) can be estimated as:

$$ R_{\text{cam}} = \frac{{D \times v \times 2 \times r_{\text{cam}} }}{\pi } $$

where *D* is the density in which the vertebrate host species occurs, *v* is the average day range of the species, and $r_{\text{cam}}$ is the average distance from the camera at which animals are detected.

Similarly, the encounter rate between hosts and ticks ($R_{\text{tick}}$) can be estimated as:

$$ R_{\text{tick}} = D \times v \times 2 \times r_{\text{tick}} $$

Here, we elaborate on this idea, to estimate the encounter rate between vertebrate hosts and live traps as:

$$ R_{\text{trap}} = D \times v \times 2 \times r_{\text{trap}} $$

which assumes that only one small mammal host passes a live trap in each checking interval as only one animal can be trapped and thus detected in each trapping interval. Note that the difference between the formulae is caused by the fact that ticks and live traps can detect animals passing from all sides (360°) while camera traps have a limited field of view [49]. $D \times v$ is the same for all three parameters in one site, so $R_{\text{tick}}$ can be described as a function of $R_{\text{cam}}$ or $R_{\text{trap}}$ and then substituted in the formula for $R_{\text{tick}}$ resulting in:

$$ R_{\text{tick}} = \frac{{R_{\text{cam}} \times r_{\text{tick}} \times \pi }}{{r_{\text{cam}} }} $$

or

$$ R_{\text{tick}} = \frac{{R_{\text{trap}} \times r_{\text{tick}} }}{{r_{\text{trap}} }} $$

$r_{\text{tick}}$ is unknown, but similar for both methods, so the encounter rates of the two methods can be compared as:

$$ \frac{{R_{\text{cam}} \times \pi }}{{r_{\text{cam}} }} = \frac{{R_{\text{trap}} }}{{r_{\text{trap}} }} $$

In other words, if we multiply the encounter rate from cameras $\left( {\frac{{R_{\text{cam}} }}{{r_{\text{cam}} }}} \right)$ with $\pi$ and estimate $r_{\text{trap}}$ we can compare the estimate of the camera traps with the estimate of the live traps. $R_{\text{trap}}$ would then be the number of rodents captured per trap per day.

$r_{\text{trap}}$ can be estimated using spatially explicit capture-recapture models [50]. Using a simple model (without covariates) in the R package *secr*, the effective sample area of all live traps combined can be estimated per species per site [51]. This estimation can be used to calculate the effective catching surface of each trap, and thus the radius from which rodents are ‘detected’ by the trap.

For all medium-sized to large mammals and birds, we used encounter rates obtained during a camera-trapping study described by Hofmeester et al. [49]. Briefly, images of animals were collected by camera traps (HC500, Reconyx Inc, Holmen, WI) mounted 40 cm above ground level on a tree at multiple, randomly generated locations within each 1-ha forest site. At any moment, two camera traps were deployed for 28 days rotating to 18 positions during March-November in 2013 or 2014. This resulted in a total of 9359 camera trapping days [49].

For all small mammals, encounter rates were estimated as described above using the trapping data obtained during a live-trapping study described by [32]. Briefly, we captured small mammals for three consecutive nights in each site over a period of two months in the summer (July-early September) of 2013 or 2014. We pre-baited the live traps for three nights prior to setting the traps and checked live traps six times at 12-h intervals. Live traps were placed in an 8 × 8 grid with 12.5 m intervals between traps. This resulted in a trapping density of 64 traps per hectare and 192 cumulative trap nights per site. We had sufficient data from 9 sites for bank vole and 11 sites for wood mouse to estimate site-specific $r_{\text{trap}}$ (Additional file 1: Table S1). It was not possible to estimate $r_{\text{trap}}$ for field vole and shrews due to a lack of sufficient data. Therefore, we averaged the available estimates over wood mouse, bank vole, and sites to derive a best available estimate of $r_{\text{trap}}$ for these species.

Encounter rates were used to estimate the probability where a resident animal arrives to a specific spot at a particular moment.

### Probable identity of the host species feeding a questing tick

We used the rate at which individual animals of a specific host species arrived to a field of view of the camera lens or a live trap to estimate the encounter probability for each species to quantify the most probable identity of the first host species that a questing tick encounters. In order to do this, we assumed that the average counts of individual animals were tied neither to the location of the camera trap or live trap, assured by the random and systematic placement of traps, nor to a specific moment of the day while the trap was operating. Furthermore, we assumed that each tick successfully feeds from the first host it encounters. All camera traps in this study were placed randomly in forest areas sampling all vegetation types and correcting for differences in sampling efficiency by calculating a vegetation and host species specific effective detection distance [52]. In situations where cameras are also placed in open habitat, it is advised to consider that ticks avoid open areas due to increased evaporation/reduced humidity. Moreover, rodents also avoid open areas due to higher chance of predation risk. In contrast, areas covered by dense undergrowth are preferentially selected both by rodents as well as ticks.

The probability of encounter by a particular host species to a host-seeking tick can be calculated because the encounter is expected to follow a Poisson process [39]. Thus, the probability of an event in which a tick fed on the host species *v* in the forest site *s* is equal to:

$$ \frac{{a_{sv} }}{{a_{s} }} $$

This is the encounter probability where $a_{sv}$ represents the encounter rate of host species *v* in the forest site *s*, and $a_{s}$ is the total encounter rates over the range of vertebrate host species identified in the forest site *s*.

### Association of four ungulate species to DOL, DON and DOA

We estimated the density of nymphal ticks (DON) as the number of nymphal ticks caught by using the cotton-flagging method for a total length of 1200 m^2^. As DON is estimated as count data with a variance in excess of the mean, we applied a negative binomial distribution to probe the relationship of DON to the probable identity of the host species at each forest site. Number of nymphal ticks on the cotton flag is equal to $j$ with the probability equal to [53],

$$ \frac{{\varGamma \left( {j + k} \right)}}{{\varGamma \left( {j + 1} \right)\varGamma \left( k \right)}}\left( {\frac{m}{k}} \right)^{j} \left( {1 + \frac{m}{k}} \right)^{ - k - j} $$

The symbol $m$ is the mean DON and $k$ is inversely related to the degree of tick aggregation, small $k$ indicates high aggregation. We used the likelihood ratio to test whether the mean $m$ is a constant across all the forest sites, or alternatively, the mean is linearly proportional to the encounter probability of the combined ungulate species. We calculated a *P*-value based on the deviance of the two nested models and a chi-square distribution with 1 degree of freedom. We selected the alternative model, modelling a linearly proportional increase, over the constant model when the *P*-value was less than 0.05. Numerical optimization was performed using Mathematica v.11.3. We performed analyses on the density of questing adult *I*. *ricinus* ticks (DOA) and the density of questing larval *I*. *ricinus* ticks (DOL) using the same approach.

### Association of leporid species and red fox to DON

To test the alternative hypothesis, i.e. the mean DON is correlated with the encounter probability of the leporid species (hare and rabbit), and red fox, we applied the negative binomial model to the encounter probability of leporids and red fox. We assumed an exponential relationship to ensure a positive mean DON. Again, we compared the alternative model with the simpler model using a *P*-value that was calculated as described in a preceding section.

### Co-infection analysis

Because we wanted to analyze the different TBPs as independent datasets, we first explored interactions between the different TBPs. For this, we looked at co-infection, the identification of two distinct TBPs, in a single *I. ricinus* nymphal tick. We applied a Fisher’s exact test to test for an association between pairs of TBPs. For this, we calculated the expected co-infection assuming independent acquisition of the two TBPs by multiplying the TBPs prevalence estimates and the observed density of nymphal ticks. In these and further analyses we combined infections with *B. garinii* and *B. valaisiana* to bird-associated *Borrelia* genospecies as these species have very similar ecologies, low infection prevalence, and we did not observe any co-infections. We did this to increase the power of our analysis.

### Association of host species to TBPs

DIN is a moment estimator of the average density of infected nymphal ticks and estimated by multiplying DON with NIP. We applied a log-normal generalized linear model [54] using the likelihood ratio to test whether DIN is a constant across all the forest sites, or if it changes with the encounter probability of local vertebrate species. For this, we tested a product of two encounter probabilities as a predictor for DIN for each TBP. This is because a questing tick at a forest site typically obtains three blood meals during its life-cycle, likely from the most widely present animals at least once and perhaps more. It is possible to calculate the probability that a tick during its life-cycle obtains a blood meal from one host species and a second blood meal from another host species by multiplying the host-specific encounter probabilities. StepAIC algorithm [54] was applied to identify a pair of host species. The predicting equation is shown in a horizontal axis label in Additional file 1: Figure S4. Again, we calculated the *P*-value as described previously. Dominance is here expressed as the difference in probability of two series of events in which a tick during its life-cycle obtains a blood meal from a host species.

## Results

### Tick densities at forest sites

**Fig. 1:**
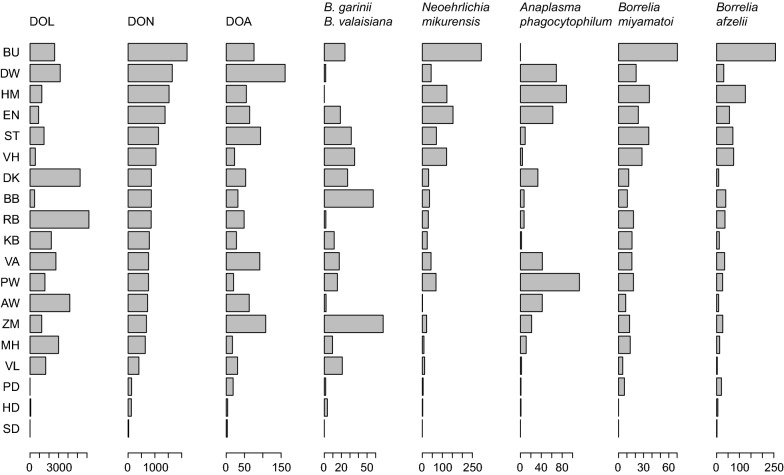
Tick densities on forest sites. Tick-borne pathogen names: density of infected nymphal ticks (calculated by multiplying the DON by the prevalence). Horizontal axis: density per 1200 m^2^ forest site. Vertical axis: two letter abbreviation per site. Locations are ranked by DON (from large to small) and displayed in Additional file 1: Figure S1. *Abbreviations*: DOL, density of larval ticks; DON, density of nymphal ticks; DOA, density of adult ticks

*Ixodes ricinus* ticks were collected at every forest site (*n* = 56,095: 992 adult, 16,568 nymphal and 38,535 larval ticks). Observed densities of ticks at the nineteen forest sites lack a clear association between the three tick life-stages (DOL, DOA and DON in Fig. 1). Densities of infected nymphal ticks (DIN) were estimated from testing individual nymphal ticks (*n* = 13,967) for the presence of TBPs (Fig. 1). TBPs were found in most forest sites but with clear differences between sites in DIN for the different TBPs (Fig. 1).

### Density of nymphal, adult and larval ticks

**Fig. 2:**
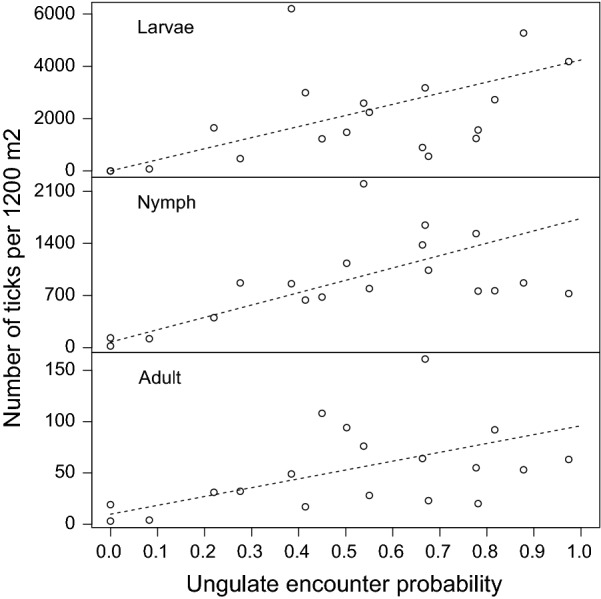
Association of four ungulate species to DOL, DON and DOA. Vertical axis: the density of ticks at each site of 1200 m^2^. Please note the different scale of the y-axes. Horizontal axis: sum of encounter rates over four ungulate species (roe deer, fallow deer, red deer and wild boar). A dashed line illustrates the best-fit negative binomial model describing DOL (*P* = 3.8 × 10^−8^, slope = 4244.54), DON (*P* = 5.3 × 10^−6^, slope = 1660.76) or DOA (*P* = 1.6 × 10^−3^, slope = 86.212)

The difference in DON between the forest sites (Fig. 2) was large: it ranged from 22 to over 2200 ticks per 1200 m^2^. The probability of encountering an ungulate at each forest site is a possible factor explaining the extent of the large difference in DON and possibly also to DOA and DOL. We detected a significant positive relationship of tick densities to the combined encounter probability of the four ungulate species (Fig. 2). No relationship to individual ungulate species, roe deer (*Capreolus capreolus*), fallow deer (*Dama dama*), red deer (*Cervus elaphus*) and wild boar (*Sus scrofa*) was identified (Additional file 1: Figure S2). The result supports a gradual and parallel increase between tick densities and the encounter probability of ungulates across the forest sites.

**Fig. 3:**
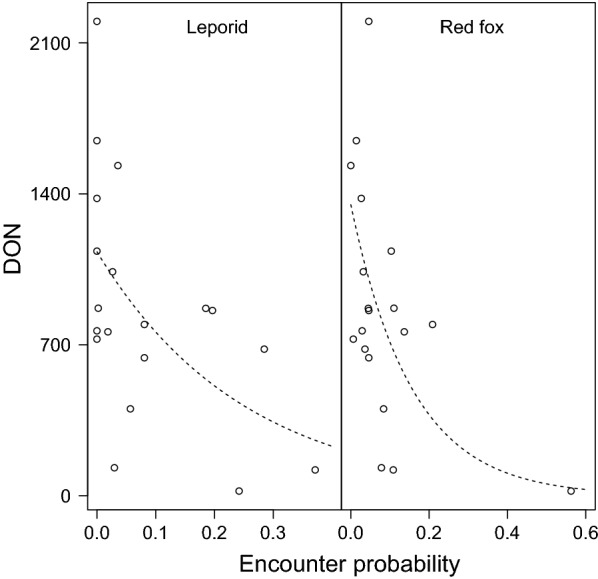
Association of leporid species and red fox to DON. Vertical axis: DON at each site of 1200 m^2^. Horizontal axis: encounter rate of leporid (the sum of hare and rabbit). A dashed line illustrates the best-fit negative binomial model (log link) describing leporid species (*P* = 2.0 × 10^−2^, exponential rate = − 3.987) or red fox (*P* = 8.8 × 10^−4^, exponential rate = − 6.396)

Separate from the ungulates, a significant negative association of DON was identified to the encounter probability of leporids, European hare (*Lepus europaeus*) and rabbit (*Oryctolagus cuniculus*), and to red fox (*Vulpes vulpes*) (Fig. 3).

### TBPs and their co-presence in nymphal ticks

The presence of two TBPs in a single nymphal tick (co-infection) was observed on average for 1 in every 16 infected nymphal ticks (3 triple co-infections, 117 double co-infections, and 1744 single infections). There are 10 possible combinations of 2 out of 5 TBPs of which we observed 8 (Table 1). Co-infection by both *B. afzelii* and *N. mikurensis* (*n* = 72) occurred significantly more than expected (Table 1). The presence of *N. mikurensis* was significantly associated with the absence of some TBPs (Table 1, *A. phagocytophilum* and bird-associated *Borrelia* genospecies). The presence of *A. phagocytophilum* was significantly associated with the absence of bird-associated *Borrelia* genospecies (*n* = 0).

**Table 1:** Number of co-infected nymphal ticks

Possible co-infection		Expected	Observed	*P*-value
*B. afzelii*	*N. mikurensis*	16.8	72	< 0.0001
*B. afzelii*	*A. phagocytophilum*	9.5	4	0.066
*B. afzelii*	*B. miyamotoi*	5.8	11	0.051
*B. afzelii*	*B. garinii/valaisiana*	3.1	0	0.079
*N. mikurensis*	*A. phagocytophilum*	26.5	5	< 0.0001
*N. mikurensis*	*B. miyamotoi*	16.3	24	0.053
*N. mikurensis*	*B. garinii/valaisiana*	8.7	1	0.002
*A. phagocytophilum*	*B. miyamotoi*	9.2	7	0.609
*A. phagocytophilum*	*B. garinii/valaisiana*	4.9	0	0.010
*B. miyamotoi*	*B. garinii/valaisiana*	3.0	2	0.772

### TBPs and their DIN

**Fig. 4:**
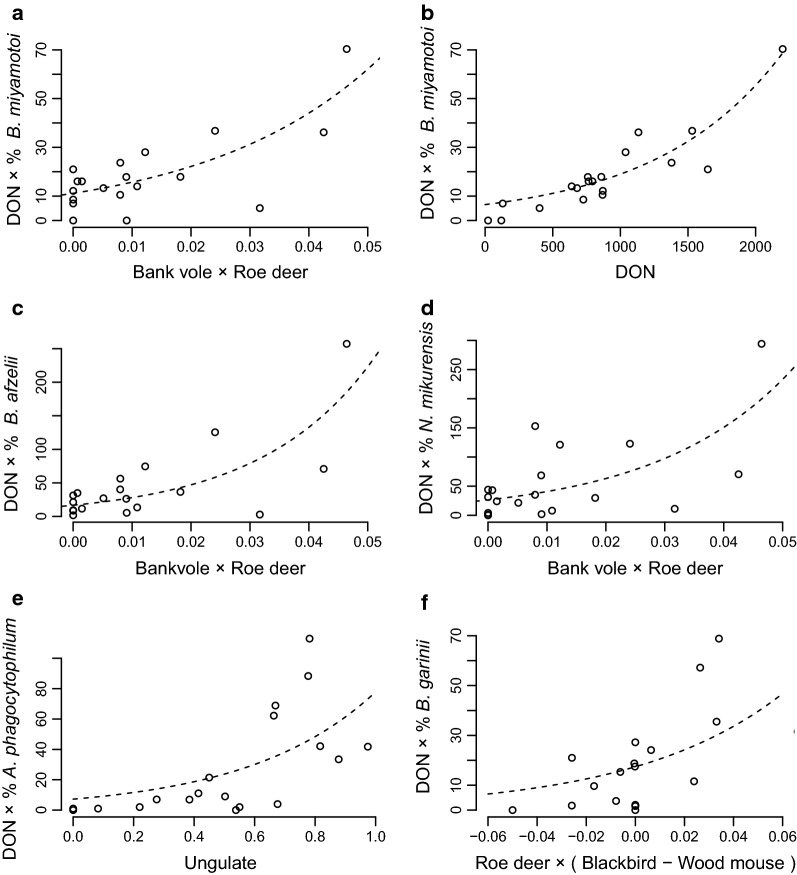
Association of host species to five TBPs. Vertical axis: DIN at each site of 1200 m^2^. Horizontal axis: a function of encounter rates except panel **b**. A dashed line in panel **a** to **f** illustrates the best-fit log-normal model describing: **a** *B. miyamotoi* (*P* = 6.7 × 10^−5^); **b** *B. miyamotoi* (*P* = 6.1 × 10^−9^); **c** *B. afzelii* (*P* = 6.4 × 10^−5^); **d** *N. mikurensis* (*P* = 1.4 × 10^−3^); **e** *A. phagocytophilum* (*P* = 2.5 × 10^−3^); **f** *B. garinii* (*P* = 5.8 × 10^−3^)

The ubiquitous presence of the investigated TBPs at every forest site is perhaps best explained by an equally ubiquitous presence of competent host species. The most widely present animal species at the forest sites were wood mouse, bank vole, roe deer and red fox (Additional file 1: Figure S3). Roe deer and bank vole correlated to a significantly positive extent (Fig. 4a, c, d) to a group of three TBPs (*B. miyamotoi*, *B. afzelii* and *N. mikurensis*). Association of *B. miyamotoi* to DON (Fig. 4b) underscores the transovarial-transmission route for *B. miyamotoi*. For *A. phagocytophilum* we found a significant positive relationship with ungulates (Fig. 4e).

Roe deer and blackbird were positively associated with high DIN of bird-associated *Borrelia* genospecies (Fig. 4f), highlighting the importance of roe deer to the tick cycle and the importance of blackbird to the TBPs cycle. Roe deer and wood mouse in contrast were associated with declining DIN for bird-associated TBPs (Fig. 4f). Dominance at each forest site of roe deer-blackbird pair over roe deer-wood mouse pair can be computed by taking the difference in the probabilities. In forest sites where a tick during its life-cycle had a higher likelihood of encountering a roe deer and a blackbird at the expense of a roe deer and a wood mouse, tended to have higher values of DIN for bird-associated TBPs (Fig. 4f). The opposite occurred when roe deer and wood mouse were dominant. The highest heterogeneity in DIN appeared to occur when the difference in encounter probabilities was zero (Fig. 4f).

Apart from the positive associations with reservoir-competent host species, we report a significant negative association of the small mammal-associated TBPs (*B. miyamotoi*, *B. afzelii* and *N. mikurensis*) to wood mouse and red fox (Additional file 1: Figure S4).

## Discussion

Here, we applied the theory of Poisson processes [39] to the encounter rates of vertebrate hosts to derive the encounter probability as a measure of the likelihood that ticks in a forest patch feed on a particular host species. To our knowledge, this is the first application of this principle to study the relationships between different host species and the number of (pathogen infected) ticks. We found that, for most TBPs, there was a positive correlation between the probability of encountering the main reservoir host and the density of infected nymphs (amplification effect). Only for bird-derived pathogens did we find a dilution effect of wood mice. This indicates that tick-borne disease risk is mainly determined by the presence of a few important reservoir hosts.

A previous analysis from the same sites reported a lack of a linear relationship between passage rates of different deer species and the density of any tick life stage [24]. The passage rate, however, depends solely on one species, whereas the encounter probability we calculated in this study depends on all host species. This difference could explain our findings that the density of all three life-stages of *I. ricinus* was linearly proportional to the probability of encounter of ungulates. The statistical methodology in the present study contributed also to some extent to the significant result. Overall, a (linear) relationship between questing tick numbers and the availability of ungulates as hosts is not an unexpected finding. The dominance of ungulates for all tick life-stages in this study suggests that long-term population dynamics of *I. ricinus* ticks share the long-term population dynamics of their ungulate hosts [55].

Few papers investigating tick burden have shown that wild boar feed very few ticks [56, 57]. A relationship to the ungulates excluding *S. scrofa* was therefore investigated by calculating the encounter probability to any of the three deer species, and testing using likelihood ratio a possibility that the three deer species are unrelated to tick densities. A significant positive relationship of the deer was detected regarding DOL (*P* = 1.45 × 10^−7^), DON (*P* = 1.56 × 10^−5^) and DOA (*P* = 0.042). In addition, a related question, whether wild boar plays a role in addition to the three deer species, was investigated by calculating the deviance of two models (the ungulates and deer) and comparing it to a chi-square distribution with 1 degree of freedom. A significantly better relationship of the ungulates compared to deer is supported (*P* = 0.0054).

Our study sites are Scots pine forest, pedunculate oak forest, or mixed forest with various vegetation covers (Additional file 1: Table S2). It is expected that the different habitats influence the tick density, and we sought to detect the link by applying Kruskal-Wallis test [54] on our dataset (Additional file 1: Table S3). No evidence for difference in tick density due to the difference in the habitat was detected regarding DOL (*P* = 0.66), DON (*P* = 0.122) and DOA (*P* = 0.20).

Previous studies into factors related to the increase in reported tick bites and Lyme borreliosis did not identify leporid densities as a potential factor [55]. A hypothesis surfacing from the present analysis (Fig. 3) is that an increase in DON in the past decades took place concomitantly with the decrease in the leporid population size in the Netherlands and elsewhere in Europe [58]. This relationship needs to be further explored as studies elsewhere in Europe have identified hares as potentially important hosts for *I. ricinus*, primarily in the absence of ungulates [59, 60]. The negative association of red fox to DON (Fig. 3) is consistent with a previous analysis of the same sites [32].

We found several associations between TBPs in co-infected ticks. The positive association between *B. afzelii* and *N. mikurensis*, also found in a previous study [14], strengthens the current understanding that both TBPs are maintained by the same reservoir hosts. Furthermore, we found significant negative associations between *A. phagocytophilum*, *N. mikurensis*, and a group of *B. garinii* and *B. valaisiana*. These findings confirm the assumption that the TBPs that we studied are maintained in separate enzootic cycles, by different host species, supporting that they can be used as independent datasets to study the role of reservoir and non-reservoir hosts in determining disease risk.

We identified roe deer to correlate with DIN regarding any of the five TBPs. This is likely because roe deer was the most common ungulate in our forest sites and an important host for adult ticks [23], thereby determining the availability of questing larvae for reservoir-competent hosts. For all TBPs, we also identified a second host species in our models, which were the reservoir-competent hosts for each of the TBPs: blackbird for bird-associated TBPs (*B. garinii* and *B. valaisiana*), and ungulates for *A. phagocytophilum*. Bank vole is a competent host for the small mammal-associated TBPs (*B. miyamotoi*, *B. afzelii* and *N. mikurensis*). Interdependence is an issue when many vertebrate species are present as in our study. Interdependence can be quantified by calculating a correlation in encounter rates between a pair of vertebrate species (Additional file 1: Figure S5).

Apart from the positive associations with reservoir-competent host species, we found a negative association between wood mouse and bird-associated *Borrelia* genospecies (Fig. 4f), which we interpret as empirical support for a direct dilution effect. Furthermore, we report a significant negative association of the small mammal-associated TBPs to wood mouse and red fox (Additional file 1: Figure S4). This could be caused by competition with and/or predation of reservoir-competent hosts as hypothetical mechanisms by which DIN can change [61]. There are, however, several other potential pathways through which encounter probabilities of different rodents and foxes could be linked, so further investigation into this topic is needed. The amount of impact, however, appears relatively mild because the events augmenting the TBP transmission and tick life-cycle by bank vole and roe deer is three times more likely to take place in a forest site compared to the reduction in numbers of wood mouse though predation by red fox (Additional file 1: Figure S4).

The analyses of our cross-sectional study did not find a support for an important role of rodents in determining the density of any of the tick life stages. One potential reason for this discrepancy is that we estimated the encounter probability of rodents based on live-trapping data. Here, we assumed that a maximum of one rodent would pass a live-trap in a 12-hour interval. It is likely that this results in underestimates of rodent encounter rates and therefore underestimates of the encounter probability of rodents. However, the bias should be similar in all areas, therefore having a minimum bias on the comparison between areas. Another option is that rodent densities are expected to fluctuate more between years than the densities of the other host species [62]. This suggests that the encounter rate in the year when we sampled hosts and ticks might be less representative of the encounter rate of hosts in the previous year (linked to DON and DIN) or two years prior to our study (linked to DOA) for rodents than for other host species. However, in a cross-sectional study design, it is not possible to assess this factor completely. Factoring the propagation host out of our cross-sectional dataset might expose a rodent. We did consider ratios between DOL/DON/DOA and attempted to identify a host species between the stages, but did not find an agreeable outcome. On ungulates many immature stages can be found, but when comparing their relative abundance to rodents, it is to be expected that rodents generally contribute more to the feeding of immature stages than ungulates [23]. In an enclosure study [24], a local tick population collapses following an exclusion of deer. It appears from our study as well, that small rodents are amply available in a natural setting and they are not a limiting factor for generating large numbers of DON and DOA.

Our proposed method, using Poisson processes to estimate which hosts are most likely to feed ticks in different communities, needs encounter rates that are comparable between species and sites. This would most easily be accomplished by using one method to estimate encounter rates for the whole host community. However, it is very hard to sample small mammals using camera traps [63]. Therefore, we used data from camera traps and live traps to estimate encounter rates and used the relative area over which the different trap types capture different species to correct absolute rates and make them comparable between species and sites [49]. Two issues that could result in biases when comparing these two capture methods are: (i) the used live traps can only capture one individual per trapping session, thereby potentially underestimating encounter rates; and (ii) the used live traps were baited (while the cameras were not) attracting animals towards the live traps, thereby potentially overestimating encounter rates [64]. Different individuals might, however, respond different to handling and bait [65]. Underestimation is a possibility at high rodent densities [66]. However, this limitation is not apparent in our live-trap dataset (Additional file 1: Figure S6). The reasoning outlined in the methods section for correcting the capture rates is, to our knowledge, the best currently available alternative. Further developing one method to estimate encounter rates for all host species, or experimental studies manipulating host encounter rates in different sites are needed in order to solve this issue.

While applying the Poisson process we made an assumption that a tick will attach indiscriminately on any host species whenever possible, e.g. failure to attach to the approaching animal is independent of host species. A violation of this assumption is certainly a viable option due perhaps to differing contact rates of hosts, tick discrimination or host reactivity to attached ticks. Nonetheless, our analysis based on the encounter events alone successfully detected a number of associations between the vertebrate community and the tick densities. The role of preferential attachment in this analysis appears limited. Blood meal analysis data is an independent source of information to validate the results of the present analyses.

## Conclusions

**Fig. 5:**
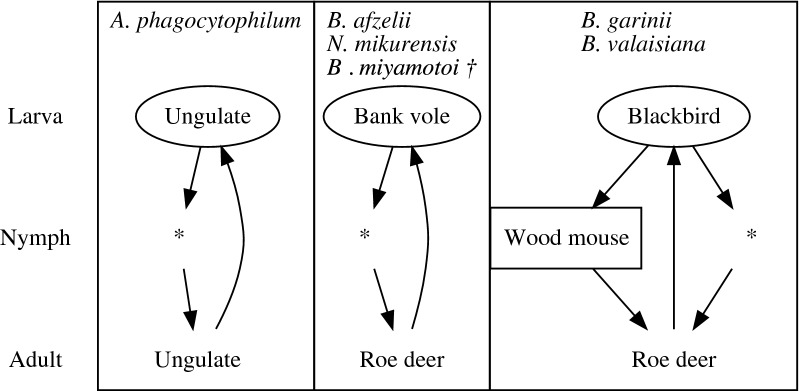
Three pathogen life-cycles identified by the analyses of camera-trapping data and the molecular analysis of pathogens. A rectangular box contains an illustration of a pathogen life-cycle. A host species is listed by the species name in a rectangular box when it is identified to maintain either the pathogen cycle or the tick life-cycle. An asterisk indicates that no particular host species is identified by the present methodology. Placement of a host species name indicates whether it is the source of the blood meal for a larval tick (top), a nymphal tick (middle) or an adult tick (bottom). A host can be either a reservoir of a pathogen (the host species name is enclosed in an oval shape), a dilution host (square) or a propagation host (name only). Arrow: Progression into the next tick life-cycle (i.e. moulting). Dagger indicates that an additional (transovarial) route is known for this TBP species

In conclusion, the results of the analyses correlate well with present knowledge and assumptions about the influence of host numbers on the DIN containing particular pathogens. We identified at least three independent pathogen life-cycles (Fig. 5), each supported by a statistical measure. Furthermore, we found that the availability of a few common host species was the main driver behind the density of infected nymphs with TPBs, supporting previous suggestions [23]. We propose the combination of estimating encounter rates using camera traps and potentially live traps, and estimating encounter probabilities of the different host species in a site as a broadly applicable pathogen-agnostic monitoring system when it is analyzed together with molecular analysis of pathogens (and hosts) in vegetation ticks. Such methodology could produce unique datasets suited to identify (candidate) reservoir host species, particularly when the identity of a reservoir host is highly uncertain. Deployment of camera traps in a green public space could be another profitable application, to quantify the risk of obtaining an *I. ricinus*-bite and potential infection with TBPs in urban areas.

## Supplementary information

**Additional file 1: Table S1.** Estimates of Rtrap for plots. **Table S2.** Characteristics and sampling effort (camera days) of the research sites. **Table S3.** Tick densities and habitat types. **Figure S1.** Nineteen forest sites in which we sampled vertebrate communities and ticks. **Figure S2.** Association of four ungulate species to DON. **Figure S3.** Presence of host species at the sampling locations. **Figure S4.** Association of four host species to TBPs. **Figure S5.** Interdependence in encounter probabilities between vertebrate species. **Figure S6.** Relationship between encounter rate, population density, and encounter probability of the two rodent species.

## Data Availability

Data supporting the conclusions of this article are provided within the article and its additional file. The datasets used and/or analysed during the present study are available from the corresponding author upon reasonable request.

## Abbreviations

TBP: tick-borne pathogens
DOL: density of questing larval *I. ricinus* ticks
DON: density of questing nymphal *I. ricinus* ticks
DOA: density of questing adult *I. ricinus* ticks
DIN: density of questing infected nymphal ticks
NIP: nymphal infection prevalence
