# Supplementary material for: Impact of vertebrate communities on Ixodes ricinus-borne disease risk in forest areas
Source: Parasit Vectors. 2019 Sep 6;12:434. doi: 10.1186/s13071-019-3700-8 (PMC6731612; doi:10.1186/s13071-019-3700-8)
Supplement: Supplementary file 1 — Additional file 1: Table S1. Estimates of Rtrap for plots. Table S2. Characteristics and sampling effort (camera days) of the research sites. Table S3. Tick densities and habitat types. Figure S1. Nineteen forest sites in which we sampled vertebrate communities and ticks. Figure S2. Association of four ungulate species to DON. Figure S3. Presence of host species at the sampling locations. Figure S4. Association of four host species to TBPs. Figure S5. Interdependence in encounter probabilities between vertebrate species. Figure S6. Relationship between encounter rate, population density, and encounter probability of the two rodent species. [file 13071_2019_3700_MOESM1_ESM.docx]

Additional file 1

Impact of vertebrate communities on Ixodes ricinus-borne disease risk in forest areas

Katsuhisa Takumi, Hein Sprong and Tim R. Hofmeester

**Table S1**. Estimates of Rtrap for plots with sufficient data to run a spatially-explicit capture recapture analysis without covariates in the secr package in R. We ran models per species per site. Rtrap was estimated based on the effective trapping area.

| Plot | Species | R-trap estimate (m) |
| --- | --- | --- |
| HM | Bank vole | 4.78 |
| VL | Bank vole | 4.96 |
| RB | Bank vole | 6.51 |
| EN | Bank vole | 7.00 |
| BU | Bank vole | 7.09 |
| VA | Bank vole | 7.28 |
| DW | Bank vole | 7.55 |
| HD | Bank vole | 7.97 |
| ST | Bank vole | 7.98 |
| VL | Wood mouse | 5.31 |
| MH | Wood mouse | 7.58 |
| HD | Wood mouse | 7.78 |
| AW | Wood mouse | 7.92 |
| RB | Wood mouse | 8.56 |
| HM | Wood mouse | 8.56 |
| VA | Wood mouse | 8.61 |
| DW | Wood mouse | 8.64 |
| PD | Wood mouse | 9.00 |
| BU | Wood mouse | 9.14 |
| VH | Wood mouse | 10.80 |
|  |  |  |
|  | R-trap Average bank vole | 6.79 |
|  | R-trap Average wood mouse | 8.35 |

**Table S2**. Characteristics and sampling effort (camera days) of the research sites

| Site | Habitat | Undergrowth vegetation^a^ | Year | Coordinates^b^ | No. camera trapping days |
| --- | --- | --- | --- | --- | --- |
| Amsterdamse Waterleiding Duinen (AW) | Mixed forest | *Calamagrostis epigejos* | 2014 | 52°20’36’’N 4°33’58’’E | 492 |
| Bergherbos (BB) | Mixed forest | *Deschampsia flexuosa* | 2013 | 51°55’14’’N 6°14’30’’E | 504 |
| Buunderkamp (BU) | Scots pine forest | *Vaccinium myrtillus* | 2013 | 52°00’56’’N 5°44’50’’E | 504 |
| Duin en Kruidberg (DK) | Mixed forest | *Calamagrostis epigejos* | 2013 | 52°26’16’’N 4°36’18’’E | 504 |
| Deelerwoud (DW) | Scots pine forest | *Vaccinium myrtillus* | 2014 | 52°05’51’’N 5°56’42’’E | 504 |
| Enkhout (EN) | Scots pine forest | *Vaccinium myrtillus* | 2013 | 52°16’25’’N 5°54’49’’E | 495 |
| Herperduin (HD) | Mixed forest | *Molinia caerulea* | 2014 | 51°45’33’’N 5°36’53’’E | 504 |
| Halfmijl (HM) | Mixed forest | *Molinia caerulea* | 2013 | 51°25’23’’N 5°19’09’’E | 504 |
| Kremboong (KB) | Pedunculate oak forest | *Dryopteris dilatata* | 2013 | 52°45’13’’N 6°31’16’’E | 504 |
| Maashorst (MH) | Mixed forest | *Deschampsia flexuosa* | 2014 | 51°42’44’’N 5°35’24’’E | 504 |
| Pettemerduin (PD) | Pedunculate oak forest | *Polypodium vulgare* | 2014 | 52°46’33’’N 4°40’19’’E | 499 |
| Planken Wambuis (PW) | Scots pine forest | *Vaccinium myrtillus* | 2013 | 52°01’54’’N 5°48’36’’E | 441 |
| Rheebruggen (RB) | Pedunculate oak forest | *Dryopteris dilatata* | 2014 | 52°46’60’’N 6°17’44’’E | 504 |
| Schoorlse Duinen (SD) | Mixed forest | *Molinia caerulea* | 2013 | 52°41’47’’N 4°40’01’’E | 504 |
| Stameren (ST) | Mixed forest | *Deschampsia flexuosa* | 2014 | 52°03’38’’N 5°21’01’’E | 486 |
| Valenberg (VA) | Scots pine forest | *Vaccinium myrtillus* | 2014 | 52°15’33’’N 5°48’47’’E | 391 |
| Vijverhof (VH) | Mixed forest | *Deschampsia flexuosa* | 2013 | 52°09’43’’N 5°13’43’’E | 507 |
| Vledderhof (VL) | Pedunculate oak forest | *Dryopteris dilatata* | 2014 | 52°52’46’’N 6°14’25’’E | 504 |
| Zwanemeerbos (ZM) | Pedunculate oak forest | *Pteridium aquilinum* | 2013 | 53°00’46’’N 6°45’19’’E | 504 |

^a^ The given plant species was the most dominant species in the herbaceous layer in the 1 hectare plot.

^b^ Coordinates given are the coordinates as measured with a handheld GPS (Garmin eTrex 20) in the middle of the 1 hectare plot.

**Table S3.** Tick densities and habitat types.

|  | DOL | DON | DOA | Habitat |
| --- | --- | --- | --- | --- |
| AW | 4186 | 726 | 63 | Mixed forest |
| BB | 468 | 869 | 32 | Mixed forest |
| BU | 2591 | 2200 | 76 | Scots pine forest |
| DK | 5274 | 870 | 53 | Mixed forest |
| DW | 3178 | 1647 | 161 | Scots pine forest |
| EN | 897 | 1379 | 64 | Scots pine forest |
| HD | 72 | 120 | 4 | Mixed forest |
| HM | 1241 | 1531 | 55 | Mixed forest |
| KB | 2239 | 794 | 28 | Pedunculate oak forest |
| MH | 2994 | 640 | 17 | Mixed forest |
| PD | 0 | 130 | 19 | Pedunculate oak forest |
| PW | 1566 | 760 | 20 | Scots pine forest |
| RB | 6203 | 859 | 49 | Pedunculate oak forest |
| SD | 0 | 22 | 3 | Mixed forest |
| ST | 1474 | 1134 | 94 | Mixed forest |
| VA | 2724 | 765 | 92 | Scots pine forest |
| VH | 555 | 1039 | 23 | Mixed forest |
| VL | 1648 | 403 | 31 | Pedunculate oak forest |
| ZM | 1225 | 680 | 108 | Pedunculate oak forest |

**
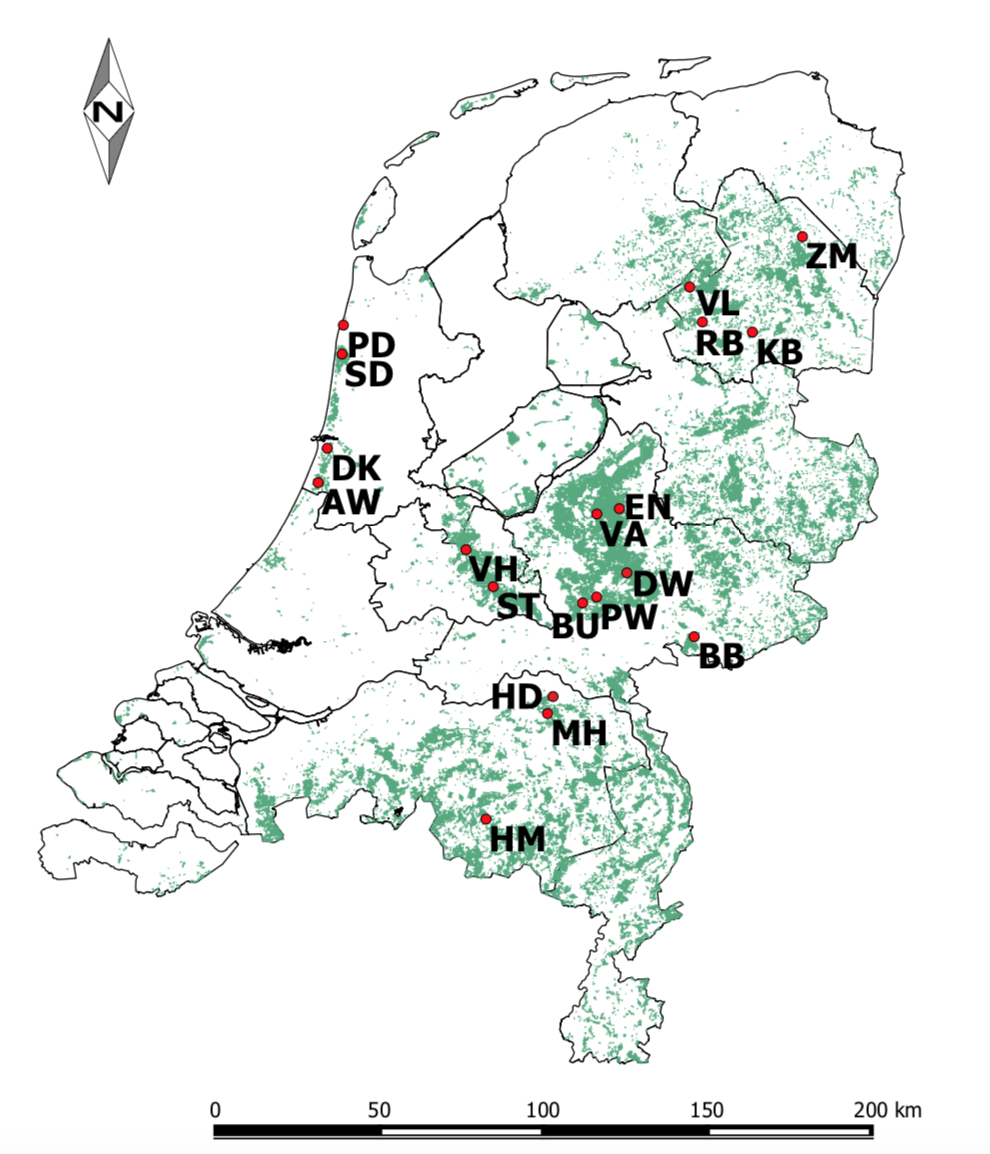
**

**Figure S1.** 19 forest sites in which we sampled vertebrate communities and ticks. Forest cover is presented in green.

**Figure S2**. Association of four ungulate species to DON

Horizontal axis: Encounter probability of individual ungulate species, or their sum. Vertical axis: Density of nymphal ticks per 1200 $m^{2}$.

**Figure S3**. Presence of host species at the sampling locations

Horizontal axis: Number of sampling forest sites. Bar height: Frequency of host presence.

**Figure S4**. Association of four host species to TBPs

Vertical axis: DIN at each plot of 1200 $m^{2}$. Horizontal axis: We calculated, the product of bank vole and roe deer encounter rates, the product of wood mouse and red fox encounter rates and the factor three, and the difference between the resulting products. The first product is on average three times greater than the second product. This resulted into the factor three.

**Figure S5**. Interdependence in encounter probabilities between vertebrate species.

A Pearson correlation in encounter probabilities between a pair of vertebrate species is calculated and it is visualized using a R-package ‘corrplot’.

**Figure S6.** Relationship between encounter rate, population density, and encounter probability of the two rodent species.
